# The Design of a Quantitative Western Blot Experiment

**DOI:** 10.1155/2014/361590

**Published:** 2014-03-16

**Authors:** Sean C. Taylor, Anton Posch

**Affiliations:** ^1^Bio-Rad Laboratories, 1329 Meyerside Drive, Mississauga, ON, Canada L5T 1C9; ^2^Bio-Rad Laboratories GmbH, Heidemann Street 164, 80939 Munich, Germany

## Abstract

Western blotting is a technique that has been in practice for more than three decades that began as a means of detecting a protein target in a complex sample. Although there have been significant advances in both the imaging and reagent technologies to improve sensitivity, dynamic range of detection, and the applicability of multiplexed target detection, the basic technique has remained essentially unchanged. In the past, western blotting was used simply to detect a specific target protein in a complex mixture, but now journal editors and reviewers are requesting the quantitative interpretation of western blot data in terms of fold changes in protein expression between samples. The calculations are based on the differential densitometry of the associated chemiluminescent and/or fluorescent signals from the blots and this now requires a fundamental shift in the experimental methodology, acquisition, and interpretation of the data. We have recently published an updated approach to produce quantitative densitometric data from western blots (Taylor et al., 2013) and here we summarize the complete western blot workflow with a focus on sample preparation and data analysis for quantitative western blotting.

## 1. Introduction

Proteomic technologies such as two-dimensional electrophoresis and mass spectrometry are valuable tools in semiquantitative protein profiling studies in order to identify broad expression patterns enabling a better understanding of molecular events, signaling pathways and mechanisms [1]. The resulting data are typically confirmed by a second, independent method such as western blotting. Western blotting was introduced by Towbin et al. [2] in 1979 and has since become a common technique used in research laboratories globally for the immunodetection and quantitation of specific proteins in complex cell homogenates. Over the past three decades, the sensitivity, robustness, and flexibility of the corresponding indicator systems have increased significantly [3, 4]. In addition, the ongoing development of detection media and reagents has provided the scientific community with ultrasensitive imaging systems giving broad dynamic range of detection enabling precise and accurate quantitation of signals from both low and high expressing proteins from the same blot. Although labs have been quick to purchase the latest detection technologies and reagents for western blotting, the associated techniques used to produce the densitometric data have not evolved leading to published data that are difficult or impossible to interpret or reproduce [5–7].

In order to obtain quantitative data from western blots, a rigorous methodology must be used as previously described [8]. Briefly, the validation of antibodies (Ab) is critical both to assure that the Ab/antigen interaction is specific and correct and to determine the dilution factor of samples that is required for protein loading in the quantitative linear dynamic range for each antibody. Furthermore, the appropriate selection of normalization method (based on reference signals obtained either by housekeeping proteins (HKPs) after immunochemical staining or total protein (TP) intensity on blotting membranes after total protein staining) must be considered to assure that the reported fold changes of the target protein are not an artifact of reference signal. Thus, data normalization is crucial to identify and correct experimental errors where reference instability becomes increasingly important with the measurement of smaller differences in target protein expression between samples [9]. The direct effect of poor normalization is evident when sample loading above 10 *μ*g of a total protein lysate per lane is required because traditional loading HKPs such as GAPDH, actin, and tubulin are grossly overloaded and therefore not serving the purpose of data normalization [8, 9]. Also, these HKPs can be affected by the treatment conditions of the experiment giving skewed results for target protein expression that do not reflect the biology of the tested samples [10–15]. Alternatively, normalization by total, blot-transferred protein has recently been shown to give excellent data for typical total protein lysates [16].

Here, we describe some general techniques to produce good quality protein samples with minimal degradation for improved reproducibility between experiments. Also summarized are the basic steps of quantitative western blotting and a standardized approach to calculating the associated densitometric data from multiple blots.

## 2. Careful Experimental Design Produces Reliable and Reproducible Data

Unlike DNA-based assays that measure a predictable type of molecule that is typically stable in a variety of conditions, proteins can vary significantly in their expression, stability, conformation, and activity under different buffer and experimental conditions. Furthermore, the presence of contaminating proteins in a homogenate can greatly affect the integrity and activity of target proteins [17]. Care must therefore be taken in the design of any protein-based assay to ensure that the apparent differences between case and control samples are not an artifact of the experimental conditions or sample handling. Factors that can have a major influence on the proteome include incubation time and temperature, as well as the parameters for processing samples such as the amount of time between tissue collection and subsequent freezing or even the conditions and timing for thawing tissue or cell pellets (Table 1).

## 3. Sample Preparation

There are several pitfalls associated with sample preparation that can directly affect the density of bands on a western blot including:

1. improper handling of tissue or cell specimens resulting in variable degradation and/or expression of proteins between samples,
2. inadequate detergents, salts, and protease inhibitors in the lysis buffer,
3. poor homogenization technique.

Since protein lysates are highly complex with contaminants such as cellular or tissue debris, fats, hydrophobic protein aggregates, nucleic acids, and proteases that can directly and negatively affect the results from western blots, it is important to use cell lysis buffers and homogenization techniques that eliminate their effects [17]. In general, homogenization buffers containing nonionic detergents such as NP-40 and Triton X-100 are less harsh than ionic detergents, such as SDS and sodium deoxycholate. Salts such as NaCl or KCl are typically added to a concentration of 100 to 150 mM to prevent protein aggregation. RIPA buffer (1% NP-40 or Triton X-100, 1% sodium deoxycholate, 0.1% SDS, 150 mM NaCl, 50 mM Tris-HCl, pH 7.8, 1 mM EDTA) and complete mini protease inhibitor cocktail tablets (Roche Applied Science) in combination with either mechanical or manual homogenization instruments have been used to produce homogenates that give solid data for western blot assays.

The proper choice of tissue homogenization technique is a prerequisite for a successful western blot assay and the method employed entirely depends on the sample type (i.e., brain versus muscle versus liver tissue as opposed to plated or suspended cells) [18, 19]. A good example of a tissue lysis protocol is as follows:

1. Snap-freeze the tissue in liquid nitrogen and then dice tissue into 1 mm pieces with a scalpel in a mortar on dry ice. Ensure that the scalpel or grinder is also frozen on dry ice to keep the cut or ground tissue close to the temperature of dry ice throughout the procedure.
2. Add the diced/ground tissue to ice-cold RIPA buffer.
3. Transfer the tissue preparation to an ice-cold Dounce tissue homogenizer (Wheaton) and Dounce 25x on ice.
4. Sonicate (Tekmar Sonic Disrupter) the Dounce-homogenized tissue on ice for 5 × 20 seconds at 50% power and clear the extracts by centrifugation at 34,000 ×g at 4°C for 30 minutes.
5. Transfer the supernatant to a new tube and perform protein assay (see below).
6. Store the supernatants at −80°C or in liquid nitrogen for long term storage.

For cell lysis, add the pelleted cells (in the case of cell suspensions) to ice-cold RIPA buffer or for plated cells, add the ice-cold RIPA buffer directly to the plate after washing the cells, and scrape and pipette the cells up and down. Continue with step (3) above.

The total protein concentration of the homogenate from either cell or tissue lysates should be measured using a detergent compatible protein assay such as the RC DC protein assay from Bio-Rad. Ideally, the homogenates would be diluted to a concentration of at least 2 mg/mL which would permit loading between 10 *μ*g and 80 *μ*g per lane of a 1 mm thick mini polyacrylamide SDS-gel.

## 4. Determine the Linear Dynamic Range of Protein Loading

**Figure 1:**
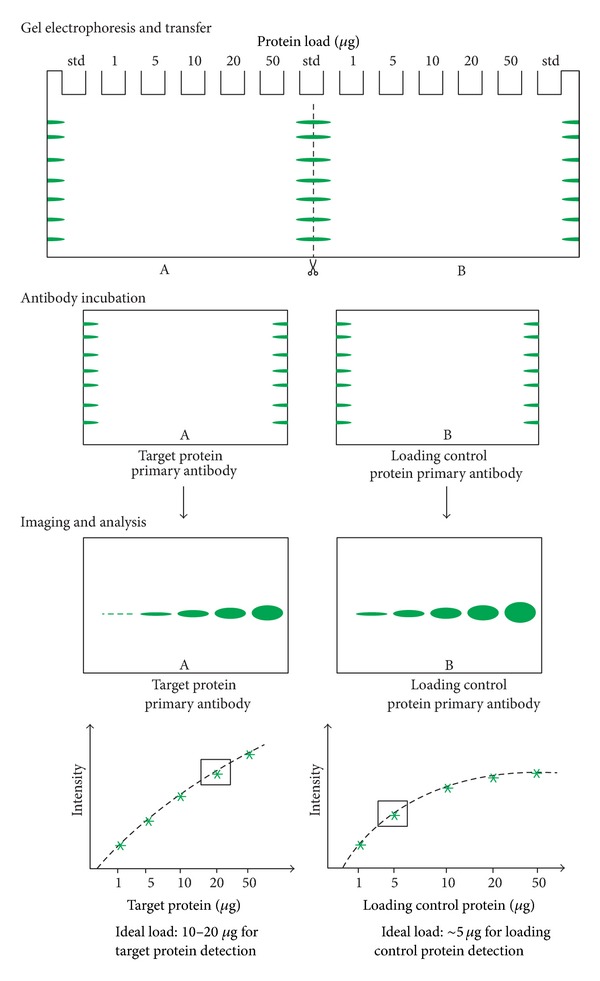
Reliable western blot data can only be generated when the proper sample amount of protein is used. Loading too much protein leads to signal saturation in western blots, yet too little produces weak signals. Once the experimental setup and conditions are established for the assay, do not change the sample load, transfer method, transfer time, antibody dilution, antibody incubation time, or temperature in subsequent experiments as these factors may significantly change the detection signals.

Most labs load a random amount of protein in each lane of a gel for western blotting that is typically between 10 *μ*g and 100 *μ*g of total lysate and there is typically no scientific basis for choosing this amount. This often results in the overloading of highly expressed, target proteins and particularly the loading controls that are used to normalize the data. This typically gives uniform band densities between lanes for the housekeeping proteins which is not due to consistent protein loading but rather from overloading the membrane with the target protein (Figure 1). To alleviate the effect of membrane saturation, a standard curve of protein load versus band density should be produced for each target protein. This can be accomplished by making a 1/2 dilution series of a pooled sample from all the lysates in the study group starting from 100 *μ*g protein load over at least 12 dilutions on a TGX stain-free SDS-gel (Bio-Rad). Stain-free detection on the ChemiDoc MP (Bio-Rad) camera system can be used to verify the loading, quality, and separation of the homogenate followed by transfer to a low fluorescent PVDF membrane using the Trans Blot Turbo (Bio-Rad) protein transfer system [8].

A typical methodology for determination of the appropriate loading for protein samples follows:

1. Transfer and blot accordingly by incubating the target protein primary antibody and associated secondary to each blot with at least four, 3-minute wash steps between each incubation.
2. Add an imager-compatible, chemiluminescence substrate such as Clarity (Bio-Rad) to develop the immunochemical signal and capture the signal using a CCD-camera-based imager such as the ChemiDoc MP (Bio-Rad).
3. Image the blots using software that provides accurate, background-subtracted densitometric tools such as Image Lab (Bio-Rad) and produce a plot of relative density versus fold dilution for each primary antibody.
4. Validate the antibodies by determining their linear dynamic range (i.e., the range in which a consistent, 1/2 decrease in density is obtained).
5. Select the protein load for each antibody that corresponds to the middle of the linear dynamic range.

**Figure 2:**
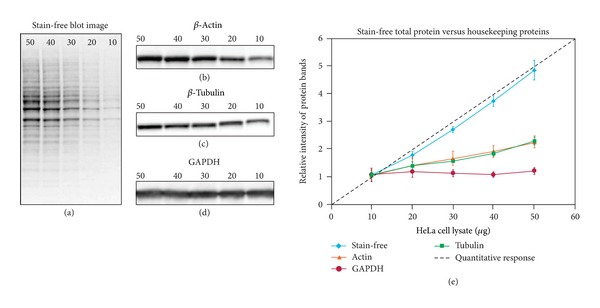
(From [9] with permission from the authors and Bio-Rad.) Linearity comparison of stain-free total protein measurement and immunodetection of three housekeeping proteins in 10–50 *μ*g of HeLa cell lysate. On the left are representative images of (a), stain-free blot and the chemi blots for (b), *β*-actin; (c), *β*-tubulin and (d), GAPDH. Lane labels correspond to total protein load (*μ*g). Although the actin and tubulin signals appear linear, the densitometric ratio was far below the predicted “quantitative response” of actual loading whereas the stain-free signal correlated to the expected result (e).

Dilution of the individual samples in the study group to the middle point in the linear dynamic range of the pooled sample for each antibody may mean that the individual protein samples require widely different dilutions for each antibody. This will assure that the densitometric data for each target protein will be within the linear dynamic (quantitative) range to give accurate and reproducible results reflecting the true biology between samples in the study set (Figure 2). Inappropriate loading of samples may result in no quantifiable difference between the samples for a given target simply due to overloading the membrane.

## 5. Determine the Appropriate Reference Signal for Data Normalization

A good reference signal or “loading control” is one that is coexpressed with the target protein within the same sample and consistently expressed between samples. HKPs such as tubulin, *β*-actin, and GAPDH have traditionally served as loading controls, but there are three potential drawbacks to using such controls.

1. HKPs may not be expressed in a uniform manner between the experimental conditions which will give erroneous results [10–15]. The same issue has been found with reference genes used for qPCR where the selection of unstable targets has led to opposite results when contrasted with stable targets [20, 21].
2. HKPs are highly abundant in lysates and have typically saturated the membrane for samples loaded in excess of about 4 *μ*g per lane (see previous section) (Figure 2). This would give these proteins the “appearance” of good normalization controls because the densities of the associated bands would all be similar between lanes as an “artifact” of membrane saturation.
3. Data normalization with HKPs relies only on one data point and provides a poor reflection of possible process inconsistencies.

Given the problems that arise with HKP controls, alternative methods for normalization have been sought out by the scientific community and we propose that an excellent loading control (LC) should meet the following criteria.

1. It has good responsiveness (1 : 1) to changes in total protein amount of individual samples.
2. It is insensitive to the influence of various physiological conditions and treatments and therefore must be quantified from the membrane itself to take into account the effect of transfer efficiency [22, 23].
3. Acquisition of the LC would ideally be possible at all phases of the western blot process (i.e., visualization of protein on the pretransfer gel lanes, posttransfer blot, and posttransfer gel lanes) thus enabling a consistent process control.
4. Acquisition of the LC must be fast, easy, and highly reproducible.
5. No lengthy process should be required for the optimization and establishment of LC.
6. The method for LC detection should be compatible with immunochemical staining.

To address these issues, the scientific community is now adopting the use of total lane density from the blot-transferred protein as a means of data normalization [24–26]. There are a number of stains that can be used to visualize, image, and quantify the transferred protein on the blot including Ponceau S, Coomassie R-350, Amido Black, MemCode, and Deep Purple. However, each of these stains has individual issues of being poorly reproducible on a day-to-day basis, limited dynamic range, and restricted compatibility with blotting membranes and immunochemical staining [25]. More recently the Stain-Free technology (Bio-Rad) has been introduced [24–26] and meets all six of the criteria mentioned above for a good LC with a linear dynamic range between about 10 and 80 *μ*g of total protein load from a typical cell or tissue lysate [8, 9]. This permits the use of total lane density from the stain-free blot for normalization between lanes for most western blot studies.

The technique for total lane normalization using the stain-free assay technology has been well-described but briefly it is as follows:  

- The quality of the electrophoretic separation can be verified within a couple of minutes. After UV-activation, the protein bands are visible in the gel and can be recorded with a camera system. The generated fluorescent signal remains stable over a couple of hours.
- The blot is imaged immediately after transfer to verify the transfer of protein from each lane.
- The image data from the total density of all the blot-transferred protein bands per lane is then recorded using Image Lab software by selecting a single band in each lane and stretching the band width to cover all the volume peaks in the lane profile.
- The background rolling disc is adjusted to a low value (between 1 and 5) for all the lanes to assure that only the total background subtracted density from the sum of all the bands per lane is acquired for normalization.

In addition, Stain-Free technology is compatible with both nitrocellulose and PVDF membranes and data normalization with SF blot images is based on many data points which is superior to HKPs.

## 6. Data Analysis: The Background Subtraction Problem

**Figure 3:**
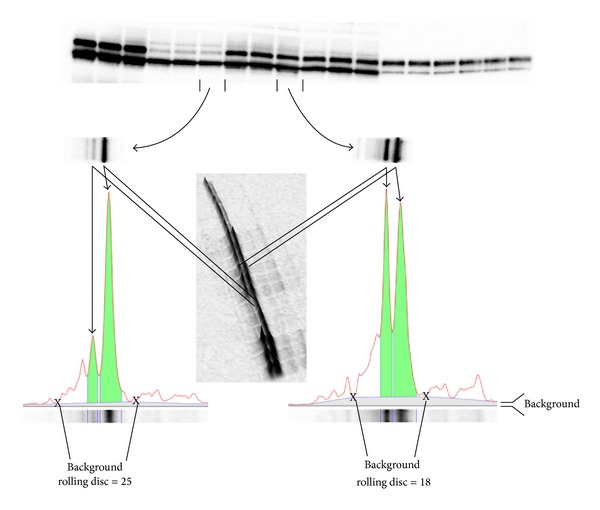
(From [8] with permission from the authors and Bio-Rad.) Image acquisition and densitometric analysis. Image Lab software version 5.0 (Bio-Rad) was used for image acquisition and densitometric analysis of the gels, blots, and film in this study. The software interprets the raw data in three dimensions with the length and width of the band defined by the “Lanes and Bands” tool in concert with the “Lane Profile” tool such that the chemiluminescent signal emitted from the blot is registered in the third dimension as a peak rising out of the blot surface. The density of a given band was measured as the total volume under the three-dimensional peak, which could be viewed in two dimensions using the “Lane Profile” tool to adjust the precise width of the band to account for the area under the shaded peak of interest. Background subtraction was set by using the rolling disc setting in the “Lanes” tool. The rolling disc values were set such that the background was subtracted under the band (i.e., peak) of interest in a uniform manner between the lanes of a given blot. In this case, the rolling disc for the two lanes analyzed was set to 18 and 25, respectively, such that the peaks of interest were cut at a consistent level between the markers shown with an “X”.

Background subtraction is a common reason to obtain variable or incorrect data from western blots [27]. Using traditional densitometric analysis methods such as volume analysis from boxed bands and background, variations in background-subtracted data can arise since the background is not subtracted from the same box in which the band resides. Furthermore, the box in which a band is selected always contains density from both the band and the associated background which becomes more prevalent with low density bands [8]. The combination of these factors can result in highly variable data when testing samples with a differentially expressed target protein using a nonspecific antibody with high background. A good alternative to volume analysis using boxes is a rolling disc background subtraction algorithm coupled with a lane profile tool (Figure 3). Image Lab software is designed with both of these tools that can be used simultaneously to ensure that the appropriate band width and lane background is selected for each lane (Figure 3).

## 7. Computational Analysis of Densitometric Data

There are numerous calculations from densitometric data using formulas buried in EXCEL spreadsheets spanning multiple worksheets and files to obtain quantitative data from western blots. It is often difficult to follow the basis of the calculations and we have found that when lab members are faced with the direct question of how to work up the raw data obtained from western blots to publishable results, there is often confusion. The analysis of western blot data can be accomplished using a very similar methodology to qPCR by calculating relative, normalized protein expression as described in the following steps (Tables 2 and 3).   

1. For each blot, multiply the background subtracted density (volume in Image Lab software) of the target protein (TP) in each lane by the ratio of density of the loading control (LC) (either housekeeping protein or total lane density) from a control sample loaded into lane 1 of all the study blots to the other lanes in the gel. This will give the normalized density to the loading control (NDL) (Table 2). The control sample is typically a pooled homogenate from all of the samples in a given study aliquoted into multiple tubes to permit the loading of a fresh control sample in lane 1 of each study blot.
2. Calculate the fold difference (FD) for each biological/technical replicate by dividing NDL from each lane by the NDL from the control sample in lane 1 (Table 2).
3. Determine the average FD and associated *P* values for the biological replicates by importing the FD from step (2) above into a statistical analysis software package such a PRISM or Analyze IT (Table 3).

## 8. Conclusions

Accurate densitometric analysis of western blots is achieved by a combination of good sample preparation, technique, detection method, software, and analysis. By following the steps outlined here, the outcome of a given experiment should produce excellent results. For highest data reproducibility and integrity the application of Stain-Free technology is highly recommended because this approach offers a novel and unique quality control tool for data normalization in a standardized manner in western blotting workflows.

## Figures and Tables

**Table 1:** Major design parameters for protein-based assays. A solid experimental design that maps the procedure, controls, replicates, experimental conditions, and sampling handling guidelines sets the foundation for production of solid, quantifiable data.

Experiment procedure	Control groups	Replicates	Experiment conditions	Sample handling
Disease or treatment groups	Time course study (i.e., *t* = 0)	Biological (difft sample per well)	Growth conditions (media and time or OD)	Precise time to harvest cells or tissues
Target proteins implicated	Normal versus disease (i.e., normal)	Technical (same sample per well)	Days of embryonic development	Sample extraction method
Potential internal controls	Untreated versus drug treated (i.e., untreated)		Amount per mass of drug or compound	Preservation method and time
These first three steps define the following: experimental parameters and the goals and the samples based on literature or experimental data typically from broad microarray or proteomics experiments			Sex, phenotype Incubation time	Thaw and homogenization procedure Total protein extraction procedure

**Table 2:** Computational analysis of densitometric data (*density data from a control sample loaded in the first lane of each gel/blot for a given study).

Biological replicates	Sample	Density (volume)			
		Target protein (TP)	Loading control (LC)	Normalized density to LC (NDL) (TP* LC/LC*)	Fold difference (NDL/NDL*)
M1	*T* = 0	8	4*	(8 × 4/4) = 8.0*	(8/8) = 1.0
M1	*T* = 1	45	5	(45 × 4/5) = 36.0	(36/8) = 4.5
M1	*T* = 2	90	6	(90 × 4/6) = 60.0	(60/8) = 7.5
M2	*T* = 0	6	5	(6 × 4/5) = 4.8	(4.8/8) = 0.6
M2	*T* = 1	40	5	(40 × 4/5) = 32.0	(32/8) = 4.0
M2	*T* = 2	88	7	(88 × 4/7) = 50.3	(50.3/8) = 6.3
M3	*T* = 0	10	4	(10 × 4/4) = 10.0	(10/8) = 1.3
M3	*T* = 1	48	6	(48 × 4/6) = 32.0	(32/8) = 4.0
M3	*T* = 2	92	6	(92 × 4/6) = 61.3	(61.3/8) = 7.7

**Table 3:** Statistical analysis of densitometric data.

FD by time	*n*	Mean	SE	Pooled SE	SD
*T* = 0	3	0.95	0.189	0.290	0.33
*T* = 1	3	4.17	0.167	0.290	0.29
*T* = 2	3	7.15	0.435	0.290	0.75

Source of variation	Sum squares	DF	Mean square	*F* statistic	*P*

Time	57.70	2	28.85	114.03	<0.0001
Residual	1.52	6	0.25		
Total	**59.22**	**8**			

Tukey contrast	Difference	95% CI			

*T* = 0 v *T* = 1	−3.22	−4.48	to −1.96	(Significant)	
*T* = 0 v *T* = 2	−6.20	−7.46	to −4.94	(Significant)	
*T* = 1 v *T* = 2	−2.98	−4.24	to −1.72	(Significant)	

## Abbreviations

Ab: Antibody
CCD: Charge-coupled device
CL: Chemiluminescence
FD: Fold difference
HKPs: Housekeeping proteins
LC: Loading control
NDL: Normalized density to the loading control
qPCR: Real-time polymerase chain reaction
SF: Stain-free
TP: Target protein
WB: Western blotting.
